# Supplementary material for: Biofunctionalized dissolvable hydrogel microbeads enable efficient characterization of native protein complexes
Source: Nat Commun. 2024 Oct 5;15:8633. doi: 10.1038/s41467-024-52948-5 (PMC11452662; doi:10.1038/s41467-024-52948-5)
Supplement: Supplementary file 1 — Supplementary Information [file 41467_2024_52948_MOESM1_ESM.pdf]

Supplementary information for manuscript

"Biofunctionalized dissolvable hydrogel microbeads enable efficient characterization of native protein complex"

Xinyang Shao[1,2]#, Meng Tian[3,4]#, Junlong Yin[3], Haifeng Duan[5], Ye Tian[2], Hui Wang[6], Changsheng Xia[6], Ziwei Wang[3], Yanxi Zhu[7], Yifan Wang[7,8], Lingxiao Chaihu[1,9], Minjie Tan[1], Hongwei Wang[3,4], Yanyi Huang[1,2,7,8], Jianbin Wang[2,3]*, Guanbo Wang[1,7]*

1 Institute of Chemical Biology, Shenzhen Bay Laboratory, Shenzhen, 518132, China
2 Changping Laboratory, Beijing, 102206, China
3 School of Life Sciences, Tsinghua University, Beijing, 100084, China
4 State Key Laboratory of Membrane Biology, Beijing Frontier Research Center of Biological Structures, Tsinghua-Peking Joint Center for Life Sciences, Tsinghua University, Beijing, 100084, China
5 CYGNUS Bioscience (Beijing) Co. Ltd., Beijing, 100176, China
6 Department of Clinical Laboratories, Peking University People's Hospital, Beijing, 100044, China
7 Biomedical Pioneering Innovation Center (BIOPIC), Peking University, Beijing, 100871, China
8 College of Chemistry and Molecular Engineering, Beijing National Laboratory for Molecular Sciences, Peking University, Beijing 100871, China
9 School of Chemistry & Materials Science, Nanjing Normal University, Nanjing, Jiangsu, 210023, China

Table of Contents:

Supplementary Figures……………………………………………………………S2-S30
Supplementary Tables……………………………………………………………S31-S33
Uncropped scans of all blots and gels presented in Supplementary Figures……………………S34

# These authors contributed equally to this work.

* correspondence to:
Prof. Dr. Jianbin Wang, E-mail: jianbinwang@tsinghua.edu.cn
Prof. Dr. Guanbo Wang, E-mail: guanbo.wang@pku.edu.cn

Supplementary Figures

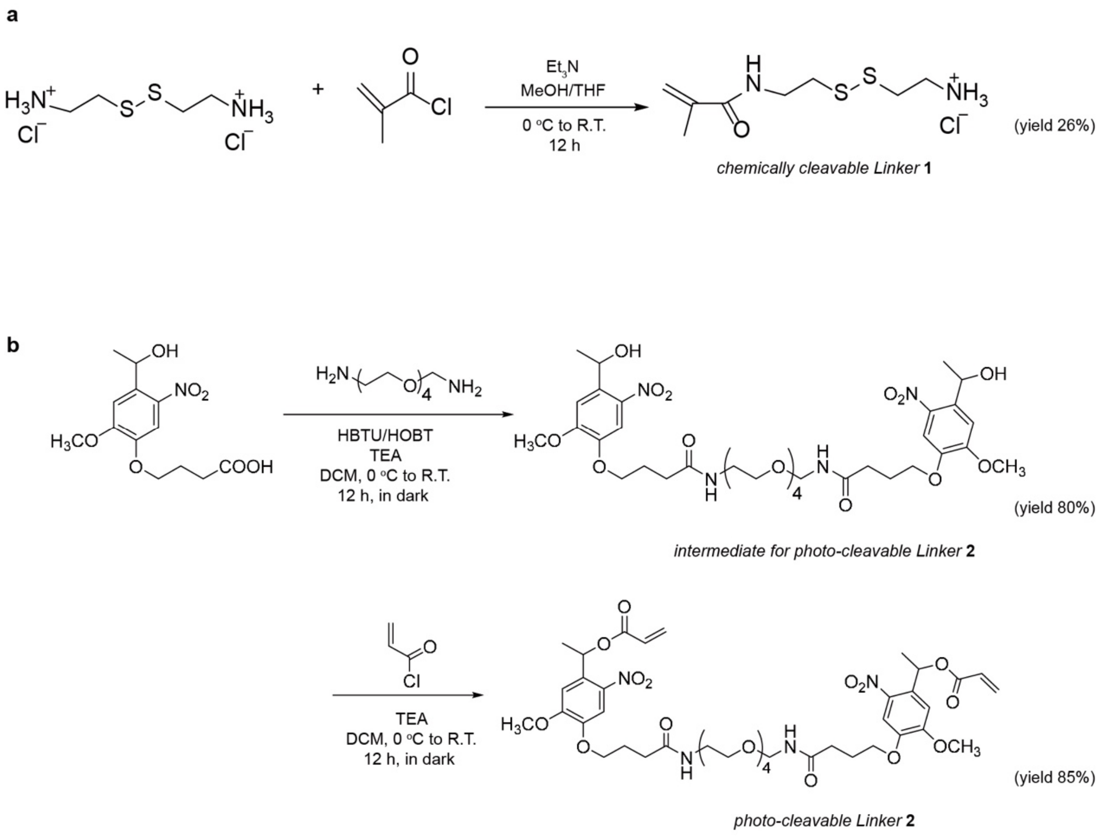

Supplementary Fig. 1 The synthesis processes of (a) chemically cleavable Linker **1**, serving as the side-chain that connects the baits to the beads, and (b) photo-cleavable Linker **2**, functioning as the crosslinker between the hydrogel polymer chains.

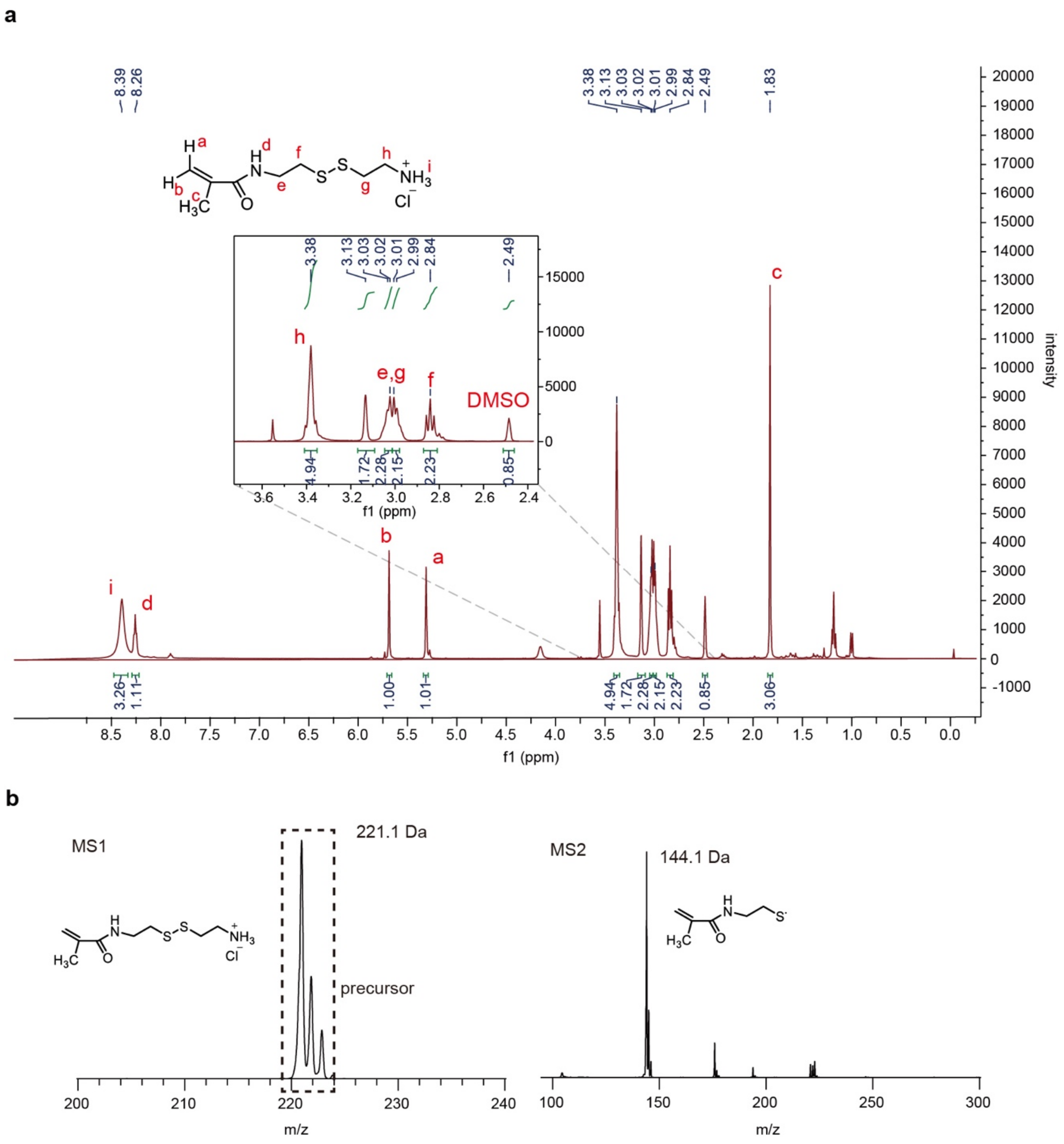

Supplementary Fig. 2 (a) ^{1}H-NMR spectrum of the synthesized chemically cleavable Linker **1** in D^{6}-DMSO. The structural formula, positioned above the spectrum, indicates the hydrogen locations corresponding to the labeled peaks. (b) Intact mass (MS1) and tandem mass (MS2) spectra of the synthesized chemically cleavable Linker **1**. The inset illustrates the structural formulae of the precursors and the primary dissociation products.

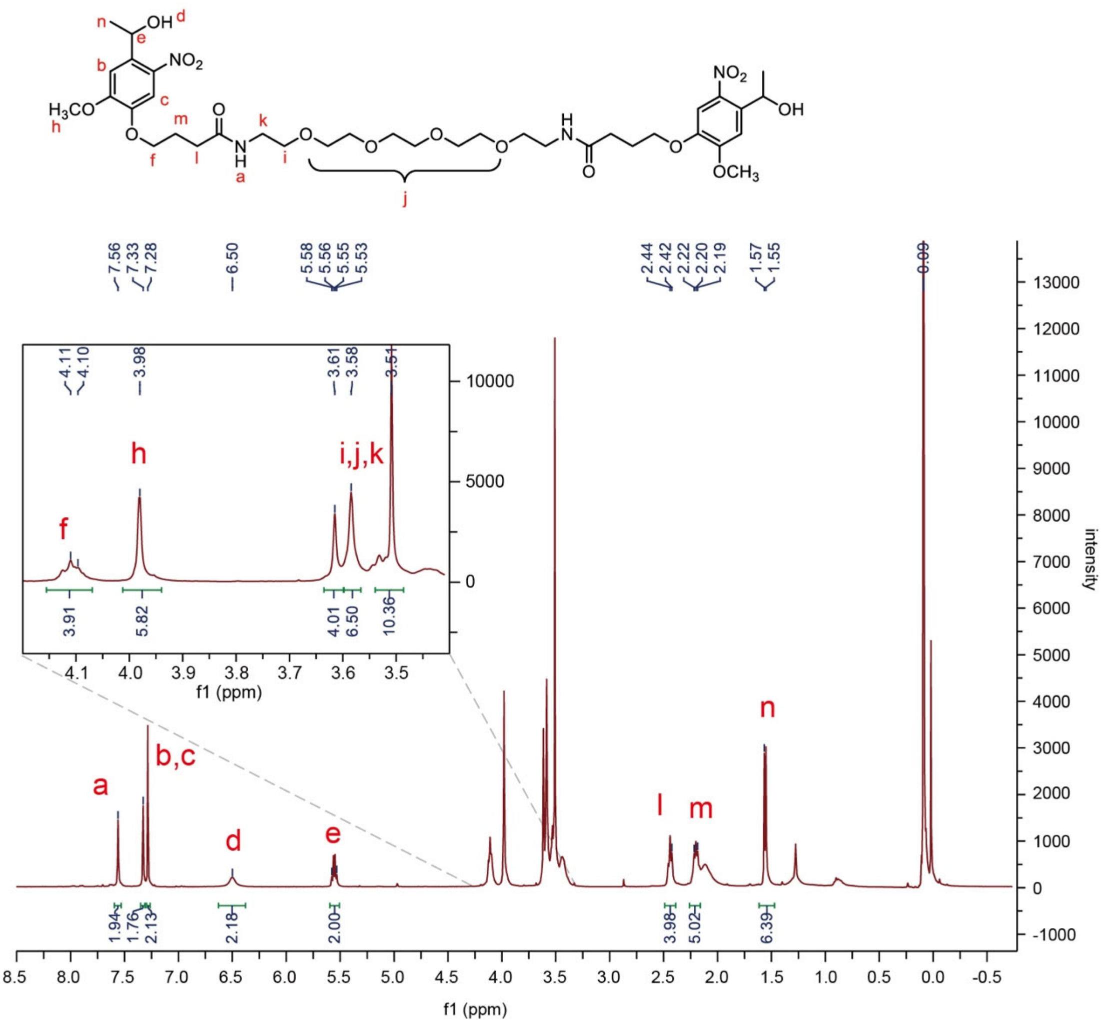

Supplementary Fig. 3 ^{1}H-NMR spectrum of the synthesized intermediate for the photo-cleavable Linker **2** in $CDCl_3$. The structural formula, displayed above the spectrum, indicates the locations of hydrogens corresponding to the labeled peaks.

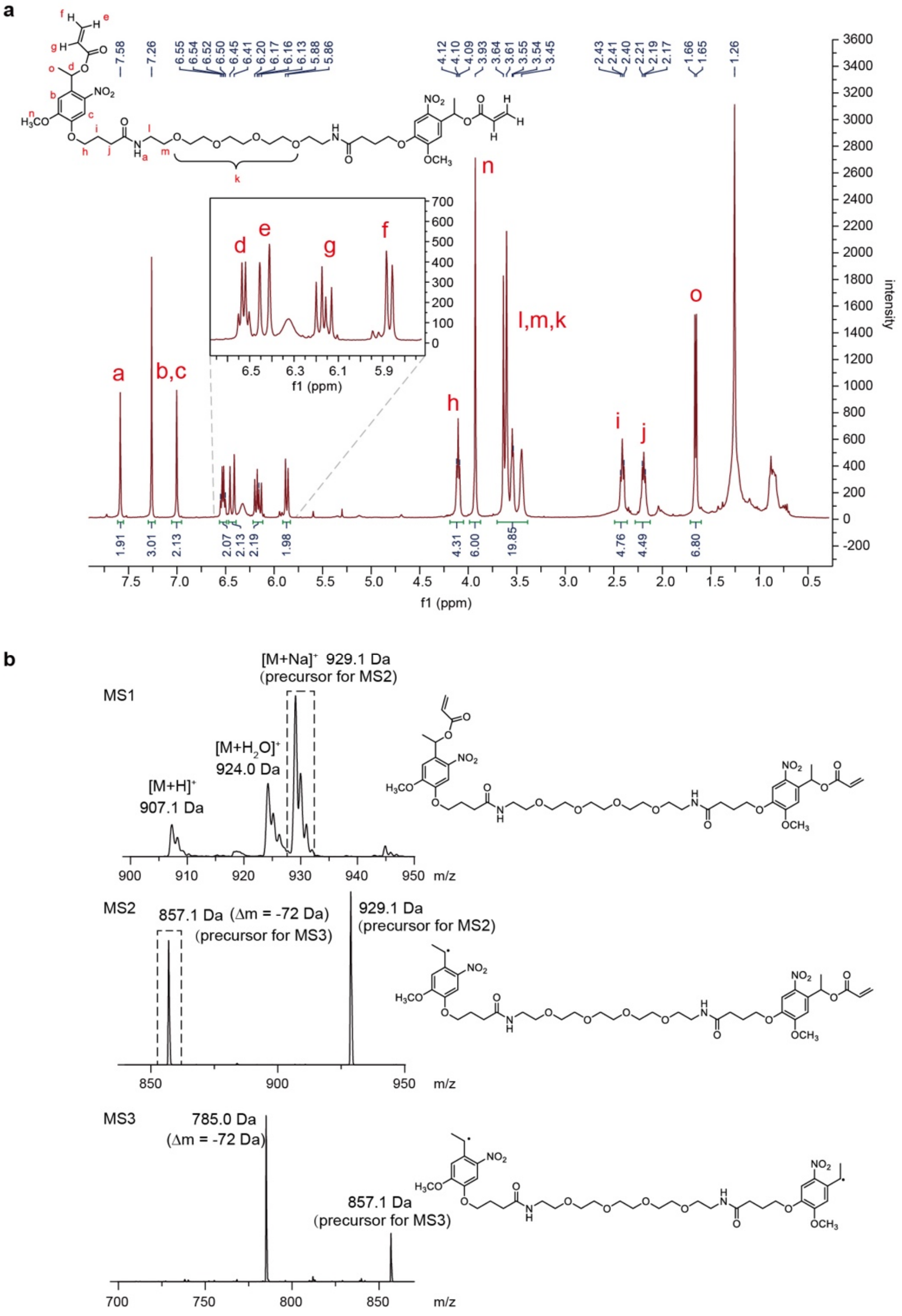

Supplementary Fig. 4 (a) ^{1}H-NMR spectrum of the synthesized photo-cleavable Linker **2** in $CDCl_3$. The structural formula, displayed above the spectrum, indicates the locations of hydrogens corresponding to the labeled peaks. (b) Intact mass (MS1) and tandem mass (MS2 and MS3) spectra of the synthesized photo-cleavable Linker **2**. The structural formulae of the precursors and predominant dissociation products are illustrated on the right.

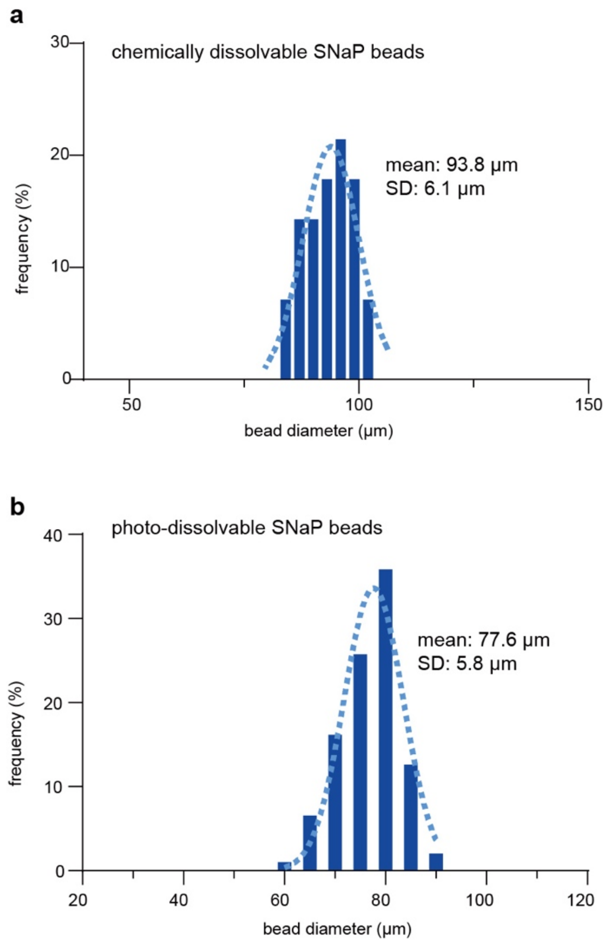

Supplementary Fig. 5 Frequency distribution histograms of the diameters of (a) chemically dissolvable SNAP beads and (b) photo-dissolvable SNAP beads, as measured through particle analysis of the fluorescence microscopic images.

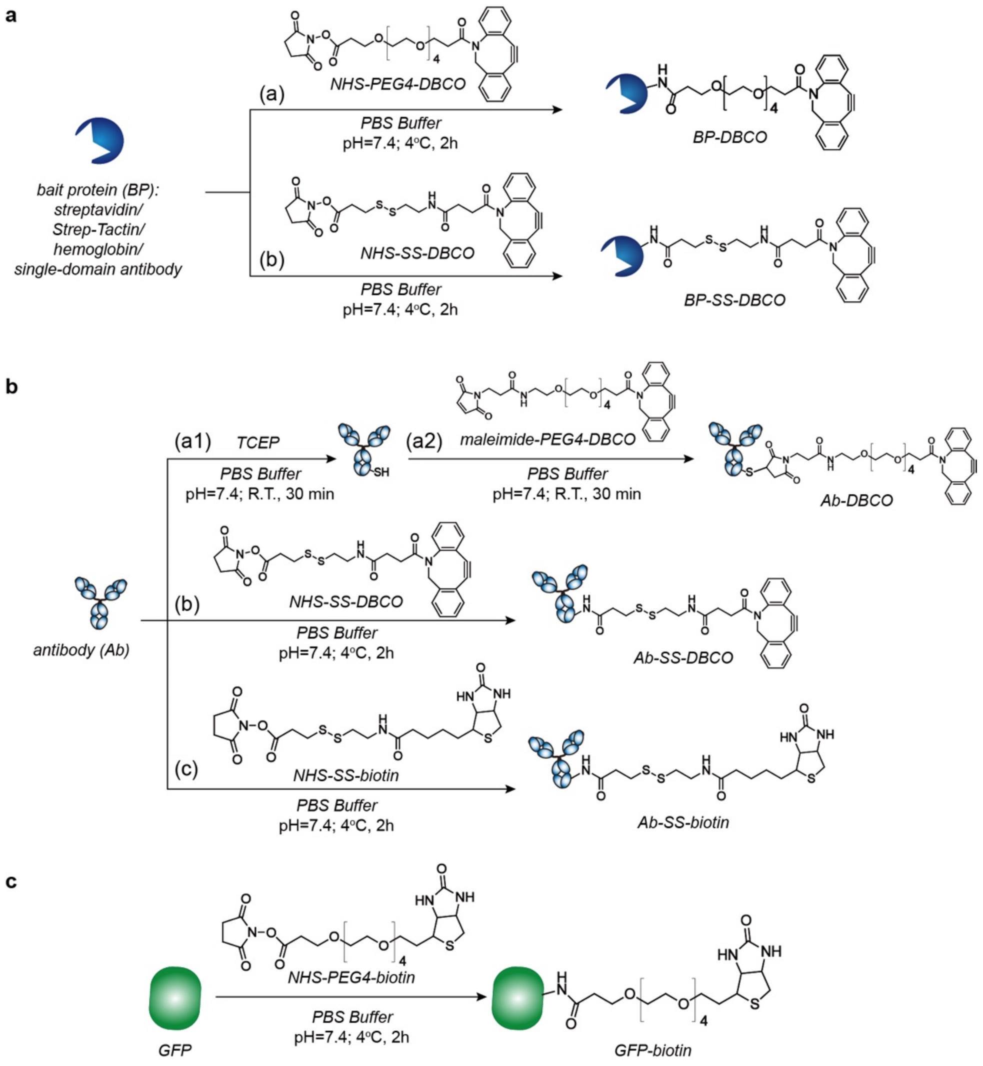

Supplementary Fig. 6 (a) Modification of bait proteins (BP) such as streptavidin, Strep-Tactin, hemoglobin, or single-domain antibody to produce BP-DBCO and BP-SS-DBCO. (b) Modification of bait antibodies (Ab) to yield Ab-DBCO, Ab-SS-DBCO, and Ab-SS-biotin. (c) Modification of target GFP with biotin for evaluate of purification performance. The term "SS" in the chemical name represents a disulfide bond.

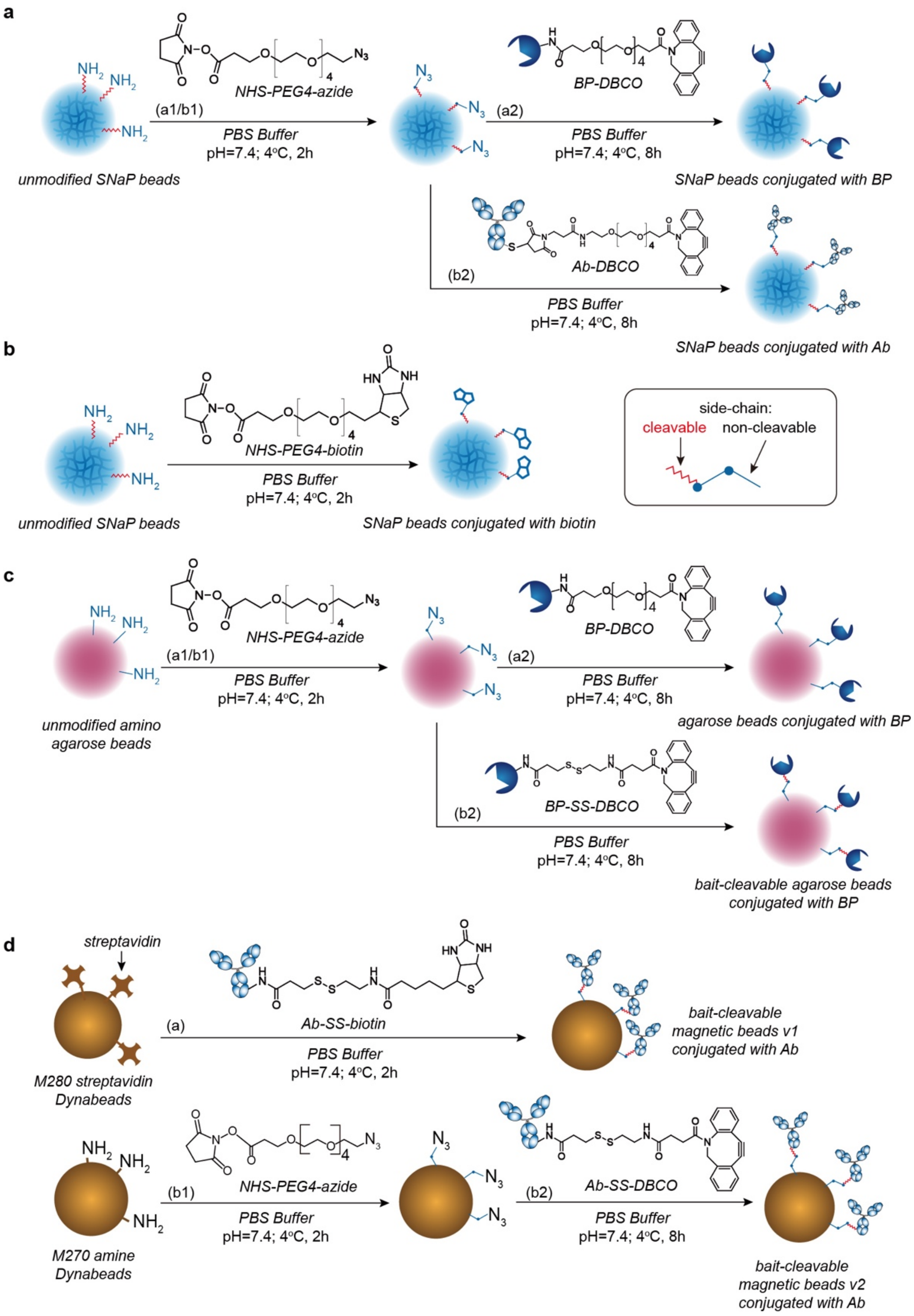

Supplementary Fig. 7 Bead modification and conjugation with modified bait proteins. (a) Modification of SNAP beads with NHS-PEG4-azide and their conjugation with BP-DBCO and Ab-DBCO, resulting in BP- or Ab-conjugated SNAP beads. (b) Modification of SNAP beads with NHS-PEG4-biotin to produce biotin-conjugated SNAP beads. (c) Modification of amino agarose beads with NHS-PEG4-

azide and their conjugation with BP-DBCO or BP-SS-DBCO, yielding BP-conjugated agarose beads and BP-conjugated bait-cleavable agarose beads. (d) Modification of streptavidin magnetic beads with Ab-SS-biotin, and modification of amino magnetic beads with NHS-PEG4-azide followed by conjugation with Ab-SS-DBCO, producing two versions of Ab-conjugated bait-cleavable magnetic beads.

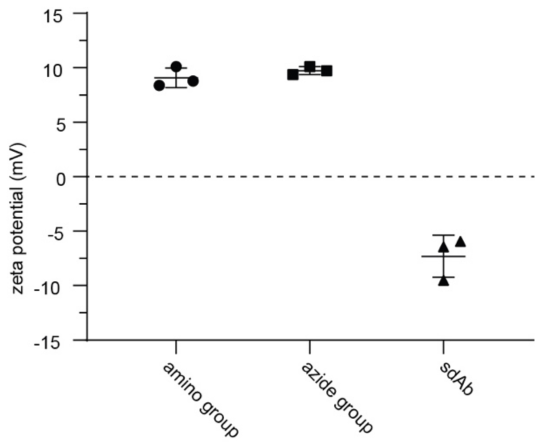

Supplementary Fig. 8 Zeta potential of the surface of SNAP beads (1) with amino groups exposed, (2) after modification with azide, and (3) following conjugation with single-domain antibody (sdAb) against GFP. Data are presented as mean ± SD of independent biological replicates in this figure.

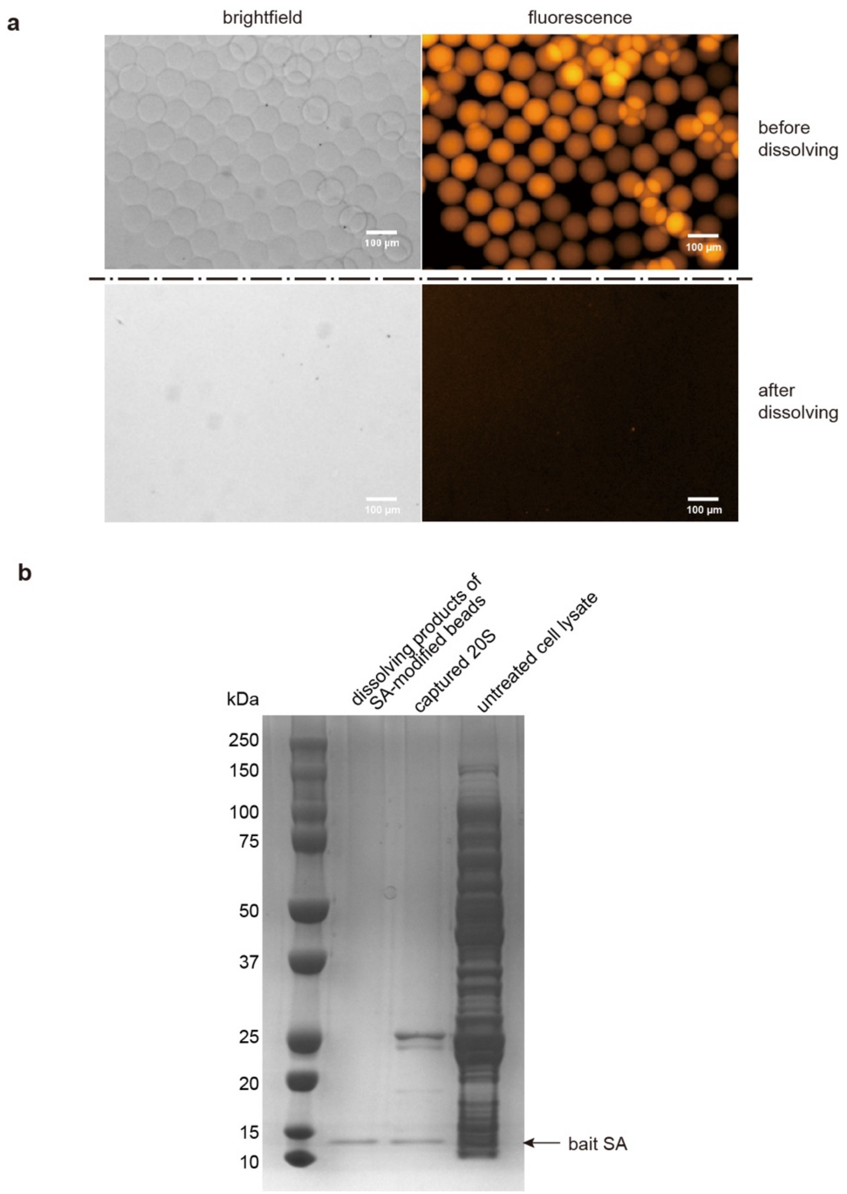

Supplementary Fig. 9 (a) Brightfield and fluorescence images of the fluorophore-labeled SNAP beads before and after the dissolving process triggered by UV-light. (b) SDS-PAGE analysis of the dissolving products of SA-modified SNAP beads, 20S purified with SNAP beads from cell lysate, and untreated cell lysate containing 20S.

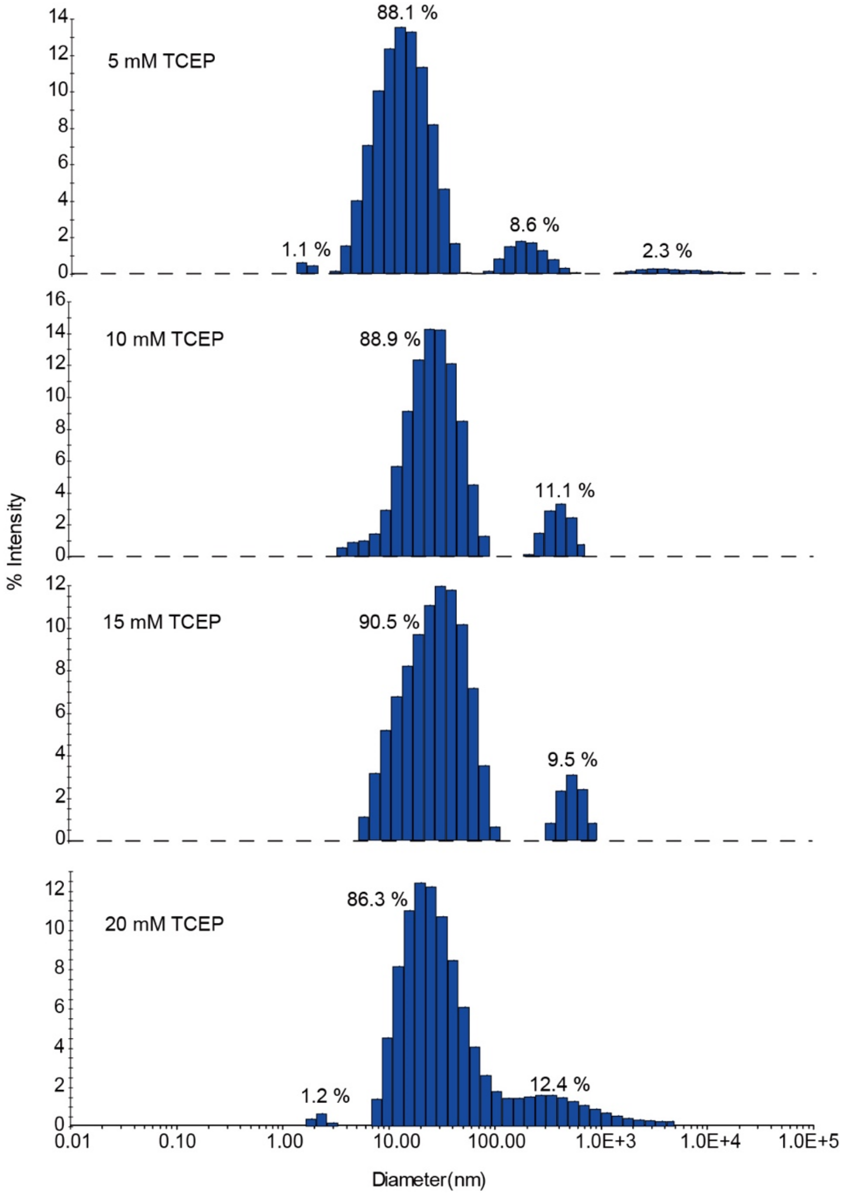

Supplementary Fig. 10 Dynamic light scattering (DLS) analysis of the dissolved beads treated with different concentrations of TCEP. The analysis suggests that TCEP concentrations exceeding 10 mM are sufficient to eliminate large-sized particles (> 1000 nm).

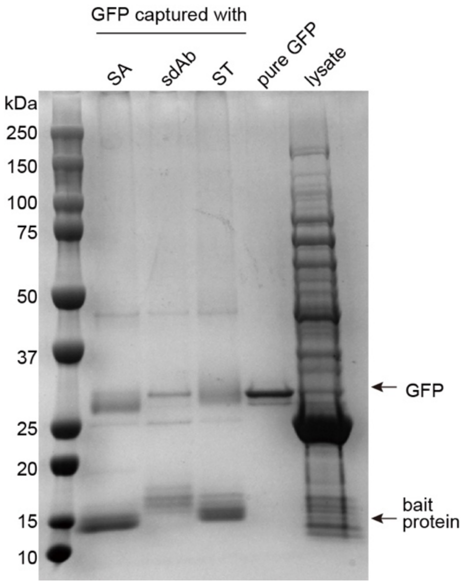

Supplementary Fig. 11 SDS-PAGE analysis of GFP samples, which were purified from *E. coli* lysate using SNAP beads conjugated with streptavidin (SA), single-domain antibody (sdAb) against GFP, and Strep-Tactin (ST). Pure GFP and untreated *E. coli* lysate samples were loaded as the controls for comparison.

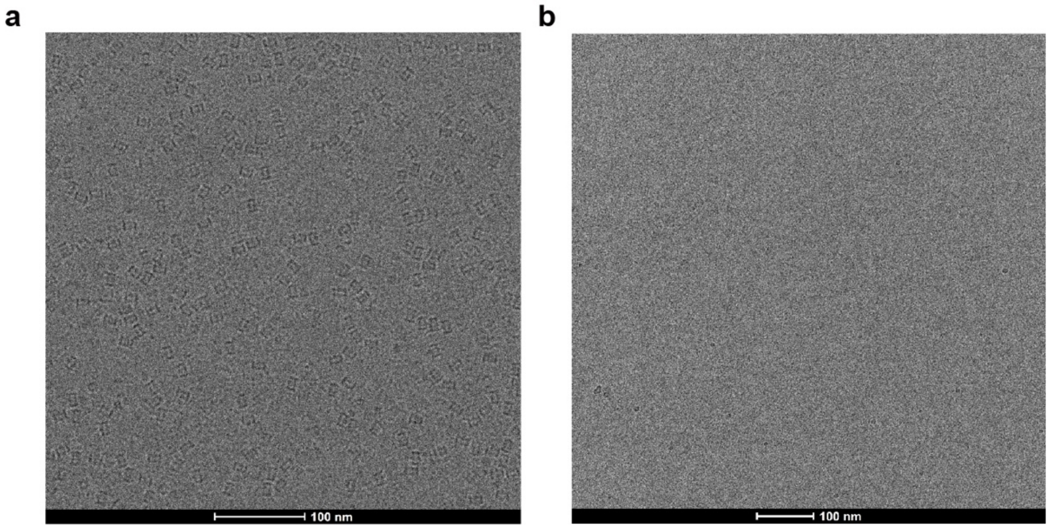

Supplementary Fig. 12 Cryo-EM images of polyacrylamide polymer chains, which resulted from dissolving of SNAP beads, in the presence (a) and absence (b) of purified 20S proteasome.

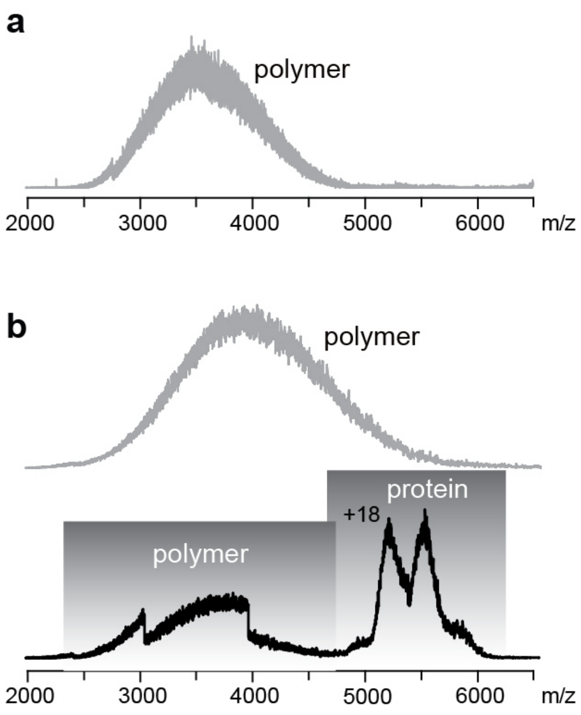

Supplementary Fig. 13 (a) Mass spectrometry profile of the products resulting from dissolving of chemically dissolvable SNAP beads. (b) Upper panel: mass spectrometry profile of the products resulting from dissolving of photo-dissolvable SNAP beads. Lower panel: mass spectrometry profile of the Hp 1-1 sample in the presence of the aforementioned dissolving products upon removal of surplus polymers through SEC. Refer to Figure S14B for the corresponding chromatogram.

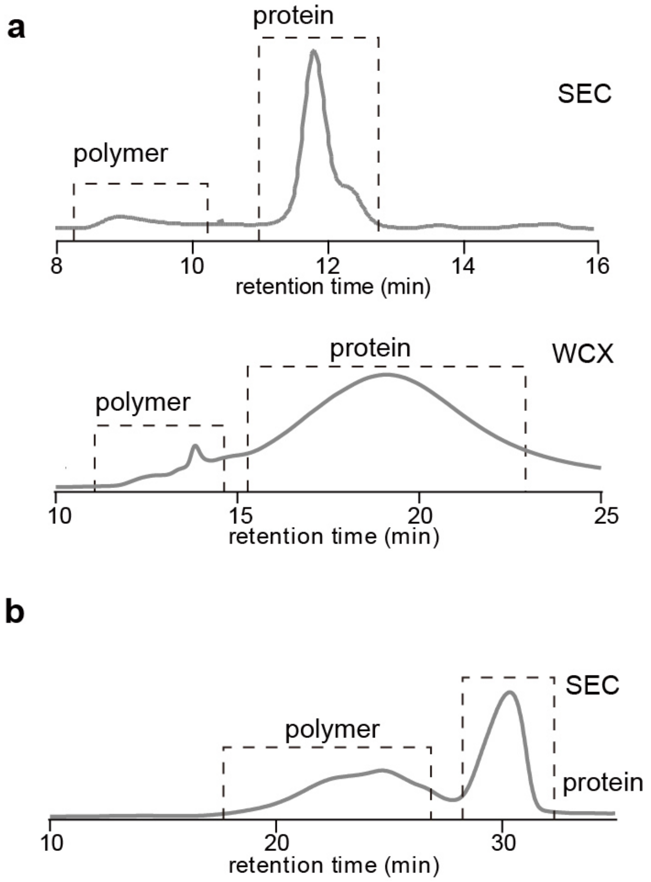

Supplementary Fig. 14 (a) Representative elution profiles demonstrating the separation of proteins, captured using chemically dissolvable SNAP beads, from the polymers resulting from bead dissolving. The top chromatogram represents size exclusion chromatography (SEC) for the GFP sample, while the bottom chromatogram represents weak cation exchange chromatography (WCX) for Hp from human serum. (b) Representative SEC elution profile illustrating the separation of Hp 1-1 from the polymers resulting from dissolving of photo-cleavable SNAP beads. All these chromatograms were acquired based on UV absorbance at 280 nm.

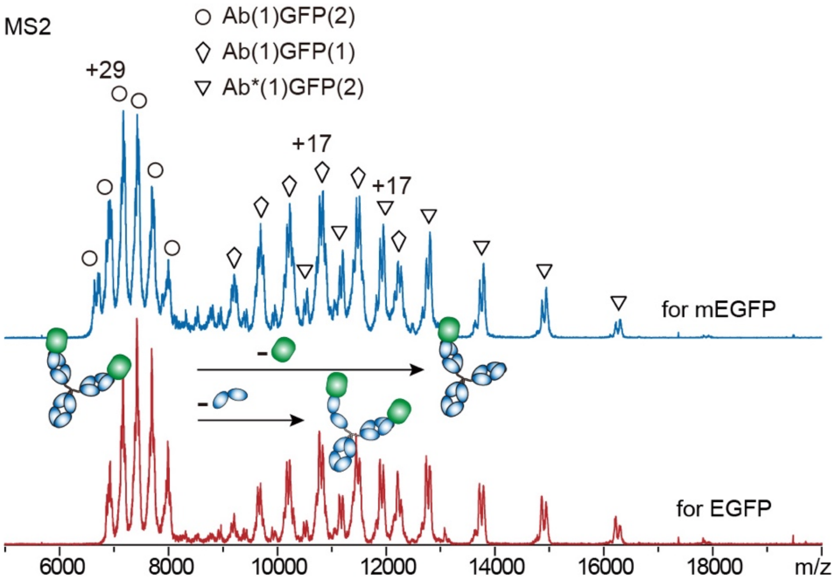

Supplementary Fig. 15 Tandem mass (MS2) spectra of the bait-target complexes, *i.e.* EGFP(2)Ab(2) (bottom) and mEGFP(2)Ab(2) (top), which were recovered from purification with SNAP beads. Collisional dissociation of the mass-selected bait-target complexes resulted in release of monomeric EGFP and mEGFP (as depicted in Figure 2G), and produced their complementary subcomplexes, as illustrated in this figure.

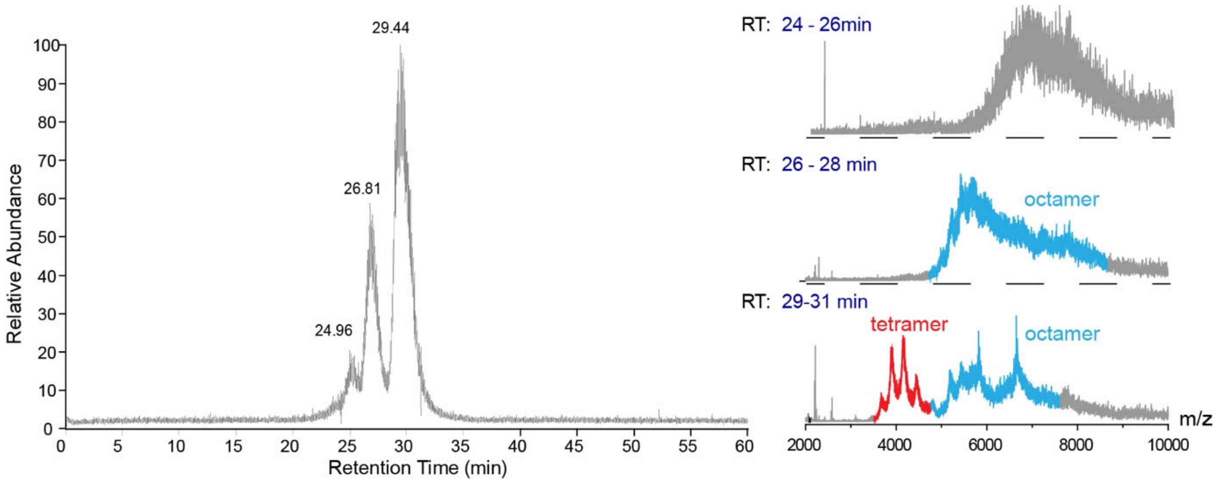

Supplementary Fig. 16 SEC profile of the avidin sample (left) and mass spectra (right) of species eluted within the indicated time windows, which were acquired in online SEC-MS. Separation and identification of different sized oligomers demonstrate the presence of larger oligomers, such as octamers, in the solution.

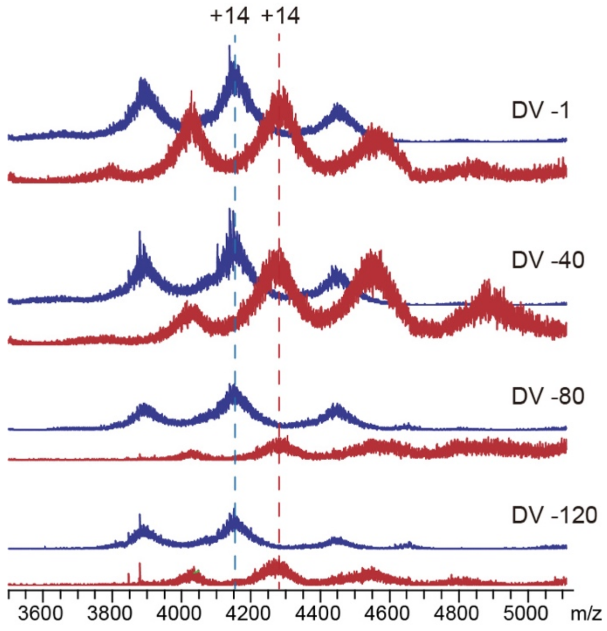

Supplementary Fig. 17 Native mass spectra of *r* avidin samples in the presence (red) and absence (blue) of the biotin bait attached to the bead-bait linkers, acquired with a constant in-source fragmentation setting of 5 and various desolvation voltage (DV) settings as indicated in the spectra.

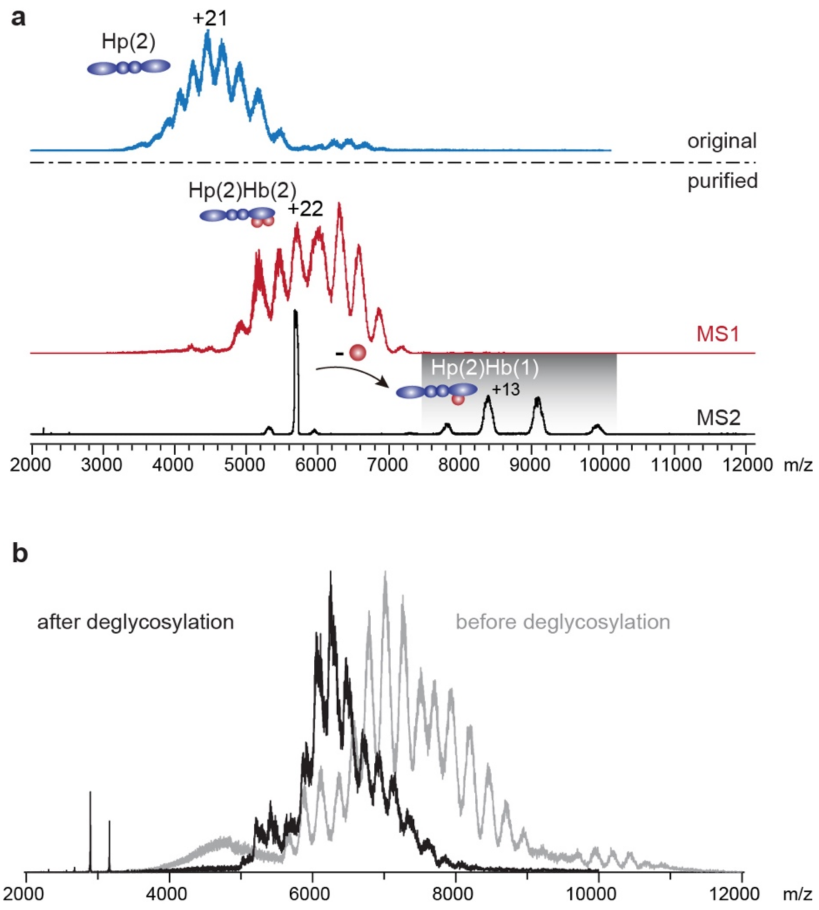

Supplementary Fig. 18 (a) Native mass spectra of original Hp (phenotype 1-1), which exist as disulfide-linked dimers (blue), and purified Hp 1-1 in complex with Hb, resulting from the chemical dissolving of SNAP beads (red). The tandem mass spectrum of mass-selected Hp:Hb complex ions in a single charge state (+22, black) validates the stoichiometry of the precursor ions and demonstrates that the integrity of both the Hp dimer and the Hp(2):Hb(2) complex is preserved under the disulfide reduction condition used for bead dissolving. (b) Native mass spectra of Hp (phenotype 2-2) before (black) and after (gray) deglycosylation treatment with PNGase F under native condition.

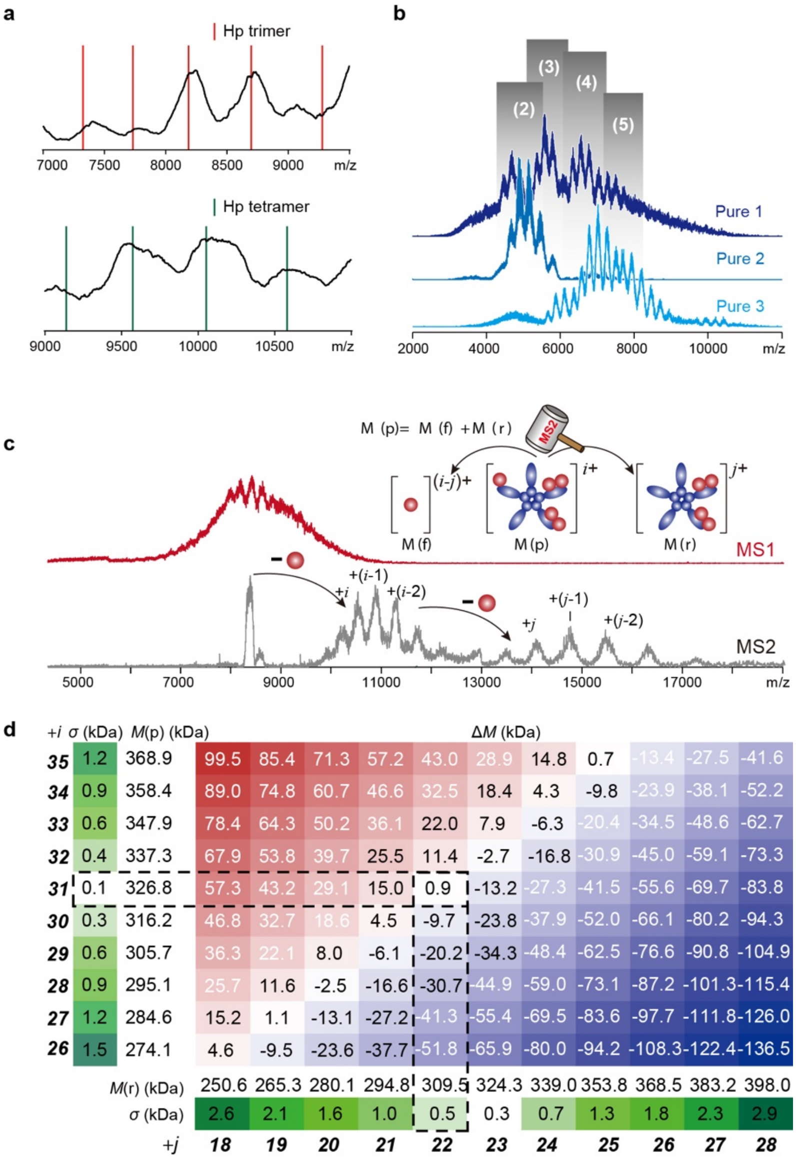

Supplementary Fig. 19 Approaches to improve the mass determination accuracy of Hp and Hp-Hb complexes. (a) Matching the detected signal peaks with droplines that indicate the theoretical m/z values of species with specific stoichiometries. (b) Masses of pure Hp samples (Pure 1: whole plasma Hp, Pure 2: Hp 1-1, Pure 3: Hp 2-2), measured by native MS, were used as the references. The numbers in parentheses indicate the oligomeric states of Hp. (c) Illustration of the tandem MS-based "fragment complementation" approach. Collisional dissociation of mass-selected precursor ions releases a specific fragment with a mass of *M*(f) and produce its complementary residual ions in a charge state distribution. The highest charge states of the precursor ions (with an average mass of *M*(p)) and the residual ions (with an average mass of *M*(r)) are denoted as +*i* and +*j* respectively. Conventional algorithms determine the value of *i* or *j* by screening for a number that leads to the least standard

deviation of the mass values calculated based on the +*i* or +*j* charge series. However, the inconsistency between m/z profiles of glycoprotein ions in different charge states (originating from different charging responses by different glycoforms) invalidates the basic assumption made in conventional algorithms[2,3], and the resulting charge assignments may deviate from the true value by a few units. Taking the mass balance of the dissociation reaction as the constraint, *i.e.* M(p)=M(f)+M(r), we listed a matrix showing the values of ΔM, which is defined as the difference between M(p) and the total of M(f)+M(r), as functions of +*i* and +*j*. The least ΔM corresponds to the correct assignment of +*i* and +*j*. Thus, the mass and stoichiometries of the analyte can be calculated based on the value of *i*. When the residual subcomplex undergoes the secondary fragmentation, it becomes the precursor of the secondarily released fragment and the secondary residual subcomplex. (d) A representative charge determination matrix showing determination of the charge state of the precursor complex ions of the secondary fragmentation as shown in (c), which were subcomplexes produced by the primary fragmentation, using the mass of the released Hb monomer as the constraint (M(f)). The mass of this complex was determined as 326.8 kDa and its stoichiometry was determined as Hp(5)Hb(5) according to the charge assignment.

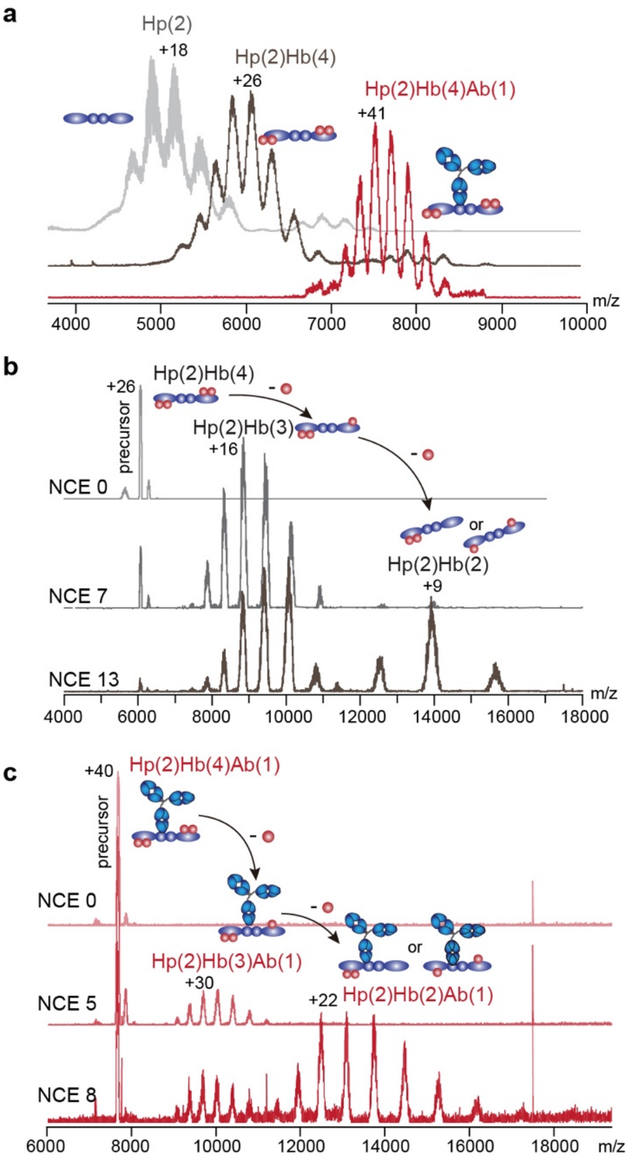

Supplementary Fig. 20 Native MS analysis of Hp and its complex with Hb bait and anti-Hp Ab. (a) Intact mass (MS1) spectra of Hp(2), Hp(2)Hb(4) and Hp(2)Hb(4)Ab(1). (b) Tandem mass (MS2) spectra of the mass-selected Hp(2)Hb(4) complex, which sequentially released Hb subunit at increased collision energies. (c) MS2 spectra of the mass-selected Hp(2)Hb(4)Ab(1) complex, which sequentially released Hb subunit at increased collision energies, without compromising the integrity of the target Hp(2)Ab(1) complex.

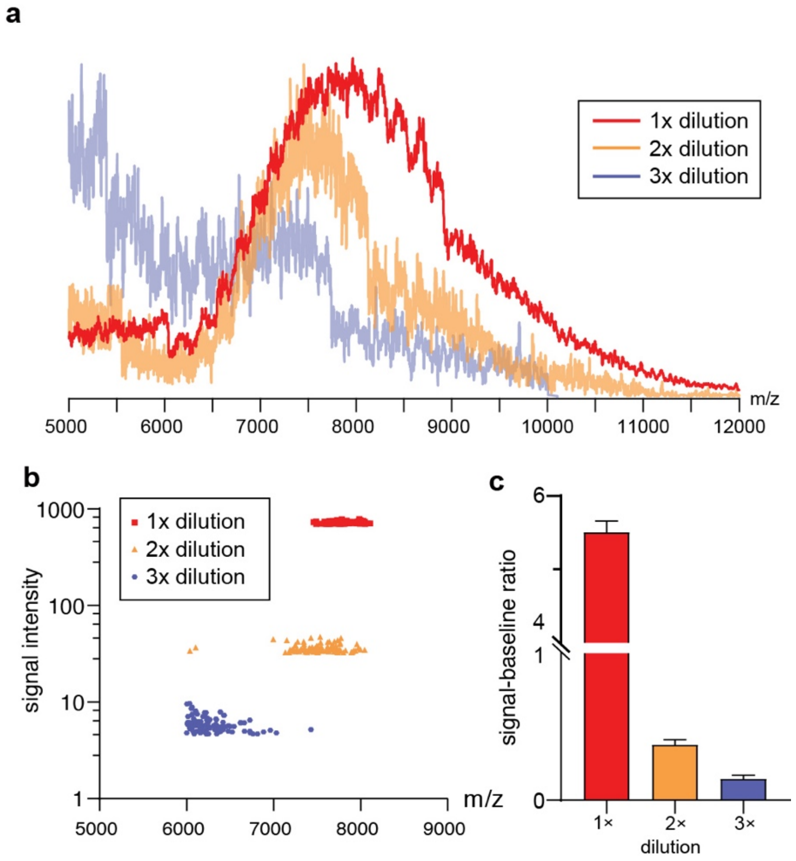

Supplementary Fig. 21 (a) Native MS spectra of Hp, purified from serum of Individual P1, at different dilution folds. (b) Signal intensities of the top 100 peaks detected at each dilution fold. (c) Signal-to-baseline ratio detected at each dilution fold. The critical dilution fold that yielded adequate spectral quality for reliable data interpretation lay between 2x and 3x. The protein concentration of the undiluted sample that experienced SNAP purification was determined to be 5.8 μg/mL using a BCA assay.

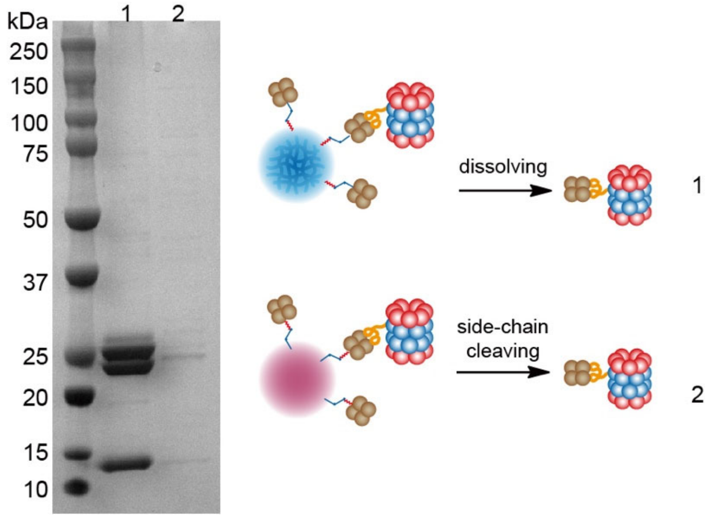

Supplementary Fig. 22 SDS-PAGE analysis of (1) 20S purified with SNAP beads that was recovered through bead dissolving, and (2) 20S purified with bait-cleavable agarose beads (fabrication process illustrated in Supplementary Fig. 7c) that was covered through cleavage of the bait-bead linker.

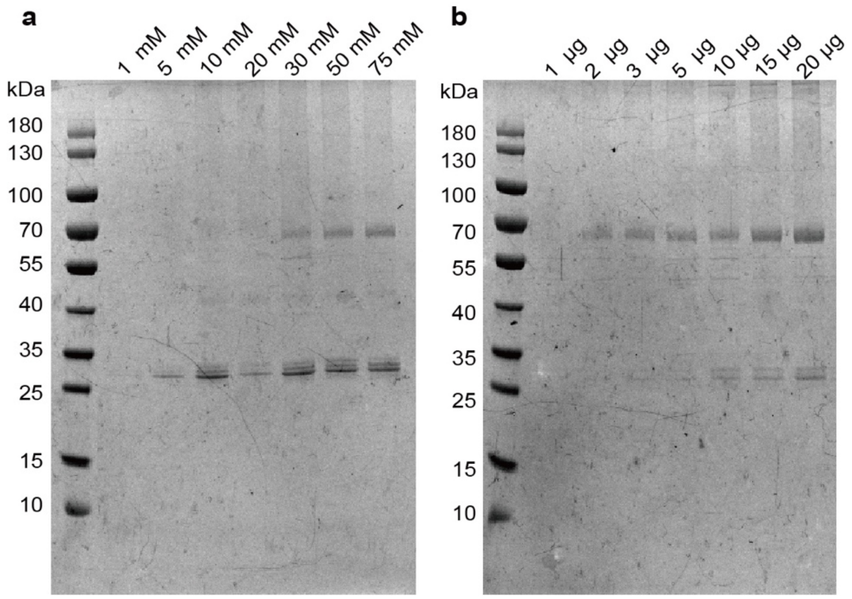

Supplementary Fig. 23 SDS-PAGE analysis of 20S proteasome samples purified by SNAP beads. (a) 20S purified using SNAP beads synthesized with different concentrations of Linker **2** (structure and synthesis shown in Supplementary Fig. 1), with an input of 20 μg ST. (b) 20S purified using SNAP beads modified with different quantity of ST bait and 30 mM Linker **1**.

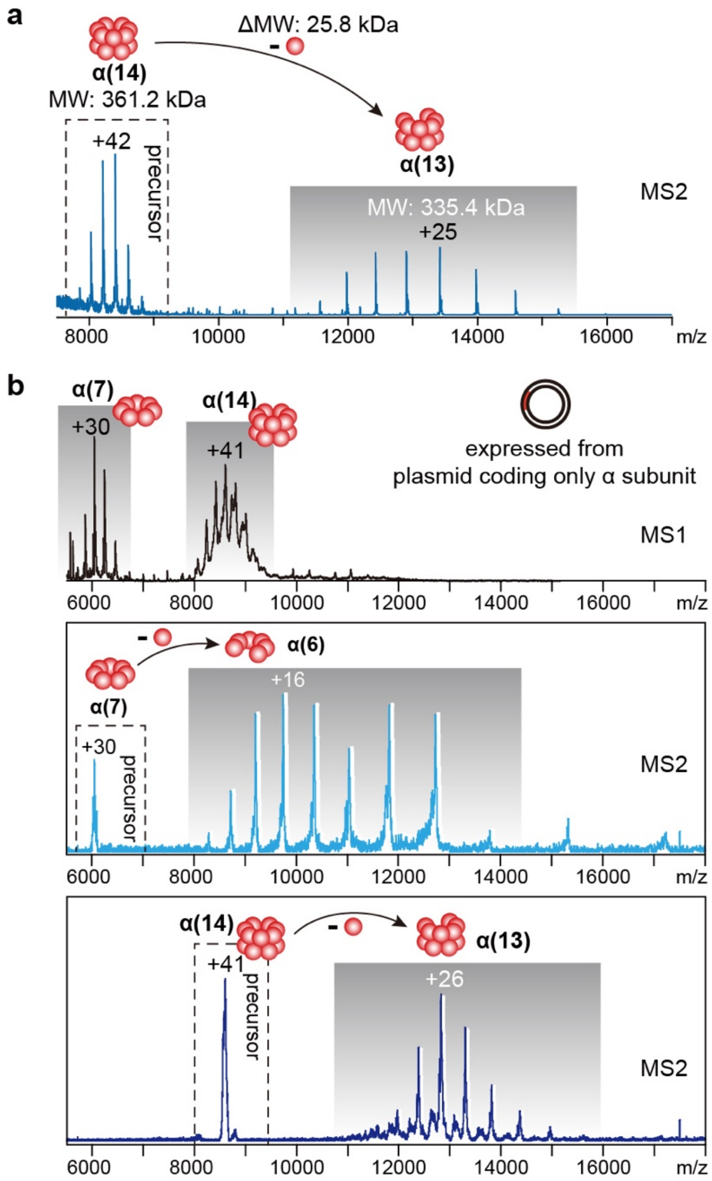

Supplementary Fig. 24 (a) Tandem mass (MS2) spectrum of the mass-selected 14-mer of α subunits, a rearrangement product of 20S expressed from two plasmids that individually encode α and β, and purified with SNAP beads. (b) Intact mass (MS1) spectrum of proteins expressed from a single plasmid that encodes only α subunit of 20S, and MS2 spectra of mass-selected heptamer and 14-mer of α subunit, which were detected in MS1. These data suggest that the heptamer and 14-mer of α subunit can spontaneously assemble in the absence of β subunit.

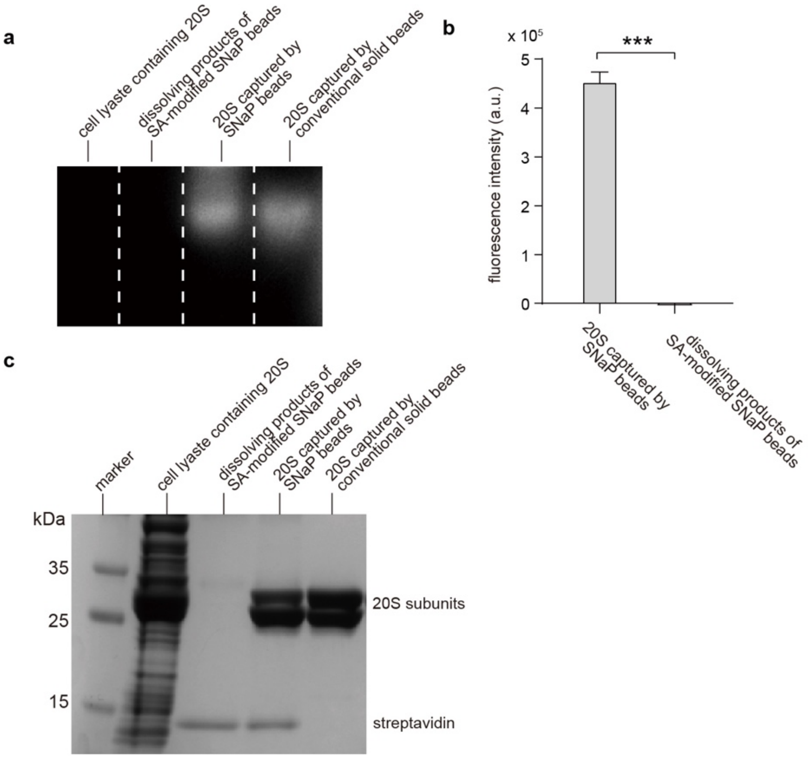

Supplementary Fig. 25 Peptidase activity assay of 20S related samples. (a) In-gel peptidase activity assay of the following samples: cell lysate containing 20S, dissolving products of SNAP beads conjugated with streptavidin (SA), 20S captured by SNAP beads, and 20S captured by conventional solid beads. (b) In-solution peptidase activity assay of 20S captured by SNAP beads, and the dissolving products of SNAP beads conjugated with SA (*** $p \leq 0.001$). Data are presented as mean ± SD of independent biological replicates in this figure. (c) SDS-PAGE of the samples loaded into the native gel shown in (a).

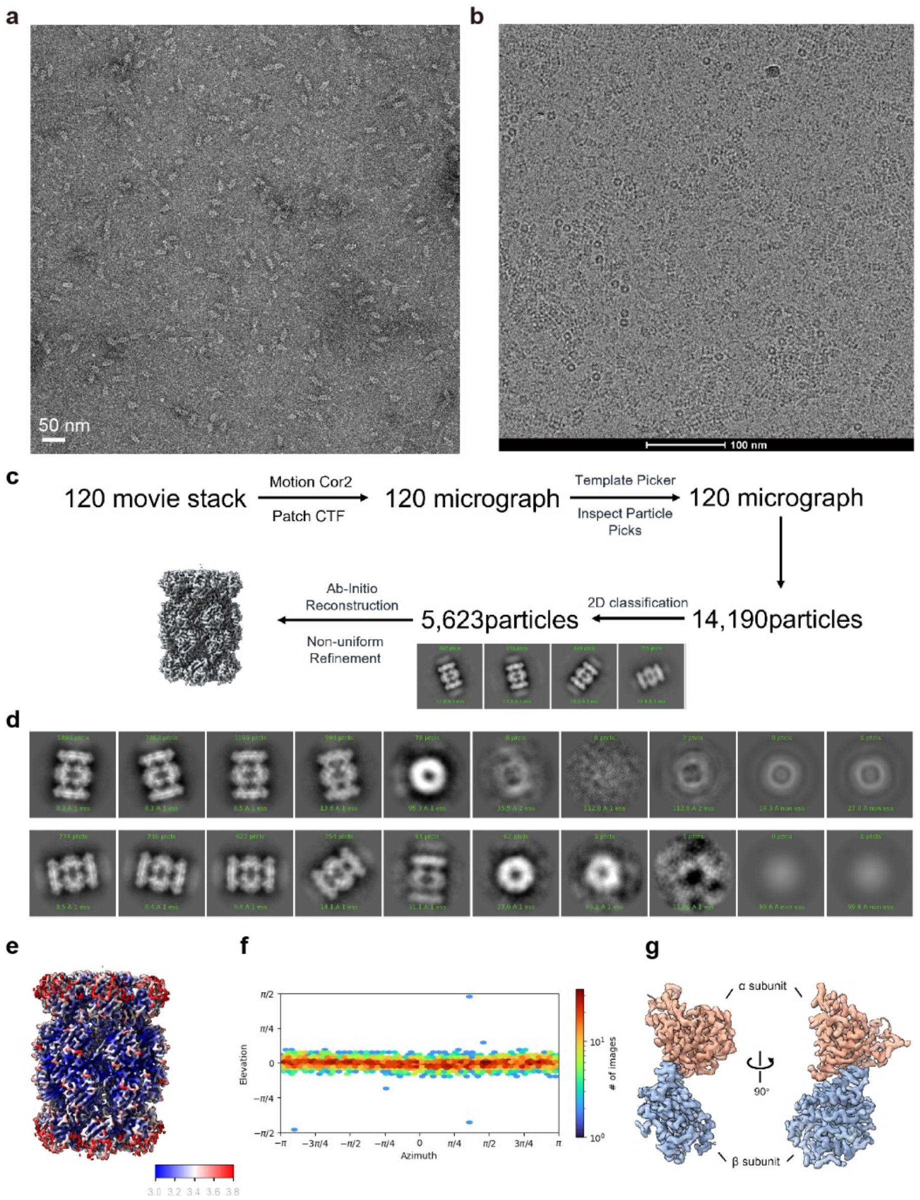

Supplementary Fig. 26 Cryo-EM characterization of SNAP-captured 20S proteasome. (a) Representative negative-stain EM images and (b) cryo-EM images of 20S purified with SNAP. (c) A schematic workflow of structural characterization using cryo-EM. (d) 2-D classification of 20S particles. (e) Local resolution estimation and (f) angular distributions in the resulting structure. (g) Different views of the segmented density map of α- and β-subunits from the density map of intact 20S.

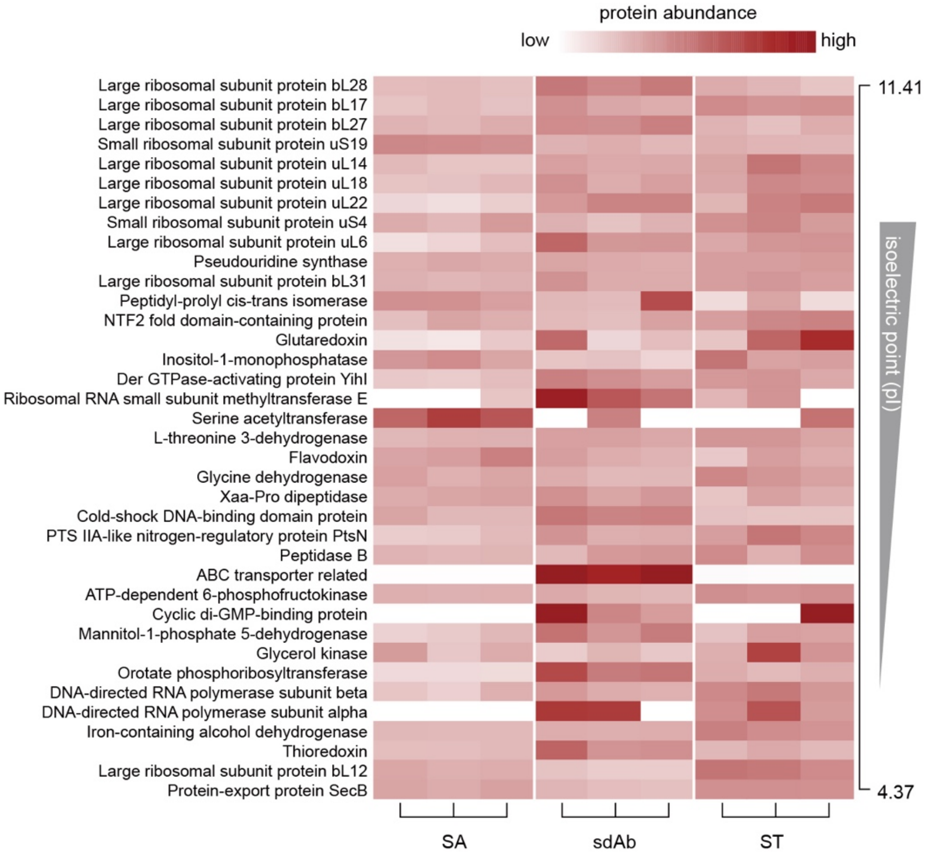

Supplementary Fig. 27 Identities and relative abundances of background proteins from *E coli* lysate detected in the output samples of GFP, which were purified with SNAP beads conjugated with SA, sdAb and ST respectively. For each type of bead, three measurements were conducted using MS-based label-free quantitative proteomics. The proteins are listed in descending order according to their isoelectric point (PI).

Supplementary Tables

Supplementary Table 1 Polymer removal methods for SNAP-purified samples in this work.

purified protein/complex	methods	filter/column specification[*]
Avidin[**]	Centrifugal ultrafiltration	30 kDa MWCO
Hp from Human sera<br>Hp 1-1<br>20S proteasome[†]	WCX	50 mm × 4 mm<br>ProPac™ Elite
GFP (sdAb bait)<br>GFP (mAb bait)[‡]	SEC	300 mm × 4 mm<br>MAbPac™ SEC-1

[*] refer to the "Liquid Chromatography" in the Experimental Section for more details

[**] including *r*Avidin and *eg*Avidin

[†] including complexes expressed from separate and single plasmids

[‡] including EGFP and mEGFP

Supplementary Table 2 Hp concentration of in blood samples from individuals P1-P4 determined using the standard immunoturbidimetry approach.

individual	Hp concentration (mg/dL)
P1	58.20
P2	112.00
P3	46.90
P4	<5.83

Supplementary Table 3 Mass values of the proteins and protein complexes identified in this work.

	ID	theoretical mass* (kDa)	measured mass (kDa)	number of charge states used for calculation
proteins	GFP released	26.9	26.9±0.0003	3
	EGFP	29.4	29.3±0.1	3
	mEGFP	29.5	29.4±0.01	4
	Hp(2)	76.9 + M(g)	92.7±0.3	4
protein complexes	Hp(2)Hb(2)	107.5 + M(g)	125.3±0.2	3
	Hp(2)Hb(3)	122.8 + M(g)	141.6±0.03	5
	Hp(2)Hb(4)	138.1 + M(g)	157.6±0.2	5
	Hp(2)Hb(2)Ab(1)	254.5 + M(g)	274.7±0.3	5
	Hp(2)Hb(3)Ab(1)	269.8 + M(g)	291.2±0.2	5
	Hp(2)Hb(4)Ab(1)	275.1+ M(g)	307.7±0.2	5
	GFP(1)Ab(1)	176.9	179.2±0.1	5
	GFP(2)AbΔLC(1)	181.3	184.1±0.1	5
	GFP(2)Ab(1)	206.3	208.3±0.2	5
	20S subcomplex: α(6)	154.8	160.0±0.7	5
	20S subcomplex: α(7)	180.6	181.1±0.4	4
	20S subcomplex: α(13)	335.4	333.1±1.2	5
	20S subcomplex: α(14)	361.2	361.3±0.6	5
	20S complex: α(14)β(14)	685.2	691.8±0.1	5

* For proteins, calculated based on amino acid sequence, excluding contribution from glycosylation, other PTMs or affinity tag; for protein complexes, calculated based on the theoretical masses of the subunits. M(g) denotes the mass of glycans.

Uncropped scans of all blots and gels presented in Supplementary Figures

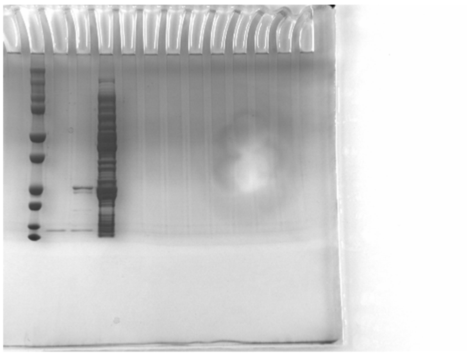

Gel scan for Supplementary Fig. 9

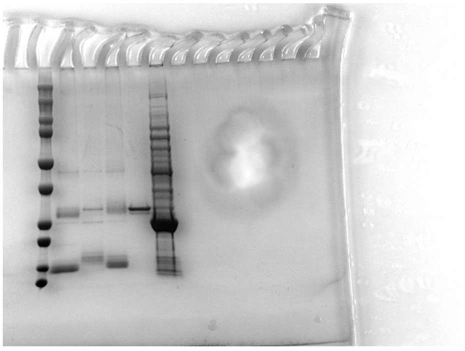

Gel scan for Supplementary Fig. 11

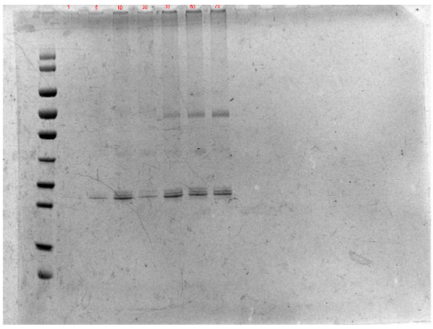

Gel scan for Supplementary Fig. 23

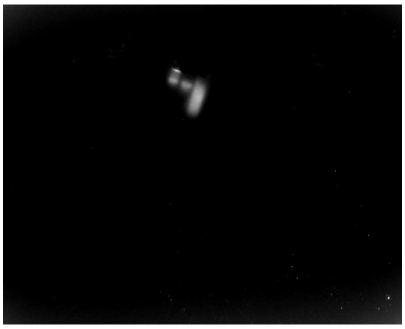

Blot scan for Supplementary Fig. 25a
(originally rotated orientation)

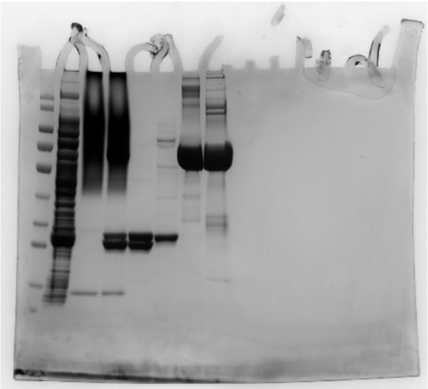

Gel scan for Supplementary Fig. 25c
