# Supplementary material for: Biofunctionalized dissolvable hydrogel microbeads enable efficient characterization of native protein complexes
Source: Nat Commun. 2024 Oct 5;15:8633. doi: 10.1038/s41467-024-52948-5 (PMC11452662; doi:10.1038/s41467-024-52948-5)
Supplement: Supplementary file 3 — Description of Additional Supplementary Files [file 41467_2024_52948_MOESM3_ESM.pdf]

Description of Additional Supplementary Files

File name: Supplementary Data 1

Description: Raw files of native mass spectrometry data presented in the main text.
