# Supplementary material for: Biofunctionalized dissolvable hydrogel microbeads enable efficient characterization of native protein complexes
Source: Nat Commun. 2024 Oct 5;15:8633. doi: 10.1038/s41467-024-52948-5 (PMC11452662; doi:10.1038/s41467-024-52948-5)
Supplement: Supplementary file 5 — Reporting Summary [file 41467_2024_52948_MOESM5_ESM.pdf]

nature portfolio

Corresponding author(s): Jianbin Wang and Guanbo Wang

Last updated by author(s): Sep 23, 2024

Reporting Summary

Nature Portfolio wishes to improve the reproducibility of the work that we publish. This form provides structure for consistency and transparency in reporting. For further information on Nature Portfolio policies, see our Editorial Policies and the Editorial Policy Checklist.

Statistics

For all statistical analyses, confirm that the following items are present in the figure legend, table legend, main text, or Methods section.

n/a	Confirmed	
☐	☒	The exact sample size (*n*) for each experimental group/condition, given as a discrete number and unit of measurement
☐	☒	A statement on whether measurements were taken from distinct samples or whether the same sample was measured repeatedly
☐	☒	The statistical test(s) used AND whether they are one- or two-sided<br>*Only common tests should be described solely by name; describe more complex techniques in the Methods section.*
☐	☒	A description of all covariates tested
☐	☒	A description of any assumptions or corrections, such as tests of normality and adjustment for multiple comparisons
☐	☒	A full description of the statistical parameters including central tendency (e.g. means) or other basic estimates (e.g. regression coefficient) AND variation (e.g. standard deviation) or associated estimates of uncertainty (e.g. confidence intervals)
☐	☒	For null hypothesis testing, the test statistic (e.g. *F*, *t*, *r*) with confidence intervals, effect sizes, degrees of freedom and *P* value noted<br>*Give P values as exact values whenever suitable.*
☒	☐	For Bayesian analysis, information on the choice of priors and Markov chain Monte Carlo settings
☒	☐	For hierarchical and complex designs, identification of the appropriate level for tests and full reporting of outcomes
☐	☒	Estimates of effect sizes (e.g. Cohen's *d*, Pearson's *r*), indicating how they were calculated

Our web collection on statistics for biologists contains articles on many of the points above.

Software and code

Policy information about availability of computer code

Data collection	The native MS data were collected with Xcalibur v 4.4.16.14 and Tune v 2.12 (Thermo Scientific). The cyro EM data were collected with serialEM v 3.17.14 and Digital Micrograph v 3.32.2403.0 (Gatan). The proteomics data were collected with Xcalibur v 4.4.16.14 and Tune v 2.12 (Thermo Scientific; for the Orbitrap system), or Compass HyStar v 6.0 (Bruker Daltonics; for the TimsTOF system).
Data analysis	The native MS data were analyzed with Xcalibur v 4.4.16.14 (Thermo Scientific); the isotopically resolved mass spectra were deconvoluted with Biopharma Finder (Thermo Scientific). The single particle EM non-uniform refinement with a D7 symmetry was performed in CryoSPARC v3.1.3. The raw LC-MS/MS proteomics data acquired with the Orbitrap and TimsTOF systems were processed with Proteome Discoverer v2.4.1.15 (Thermo Scientific) and PEAKS Studio X Pro v10.6 (Bioinformatics Solutions), respectively.

For manuscripts utilizing custom algorithms or software that are central to the research but not yet described in published literature, software must be made available to editors and reviewers. We strongly encourage code deposition in a community repository (e.g. GitHub). See the Nature Portfolio guidelines for submitting code & software for further information.

Data

Policy information about availability of data

All manuscripts must include a data availability statement. This statement should provide the following information, where applicable:

- Accession codes, unique identifiers, or web links for publicly available datasets
- A description of any restrictions on data availability
- For clinical datasets or third party data, please ensure that the statement adheres to our policy

The raw files of proteomics data generated in this study have been deposited in the ProteomeXchange Consortium (https://proteomecentral.proteomexchange.org) via the MassIVE partner repository (https://massive.ucsd.edu/ProteoSAFe/static/massive.jsp) with the identifier PXD054976 [http://proteomecentral.proteomexchange.org/cgi/GetDataset?ID=PXD054976] (in ProteomeXchange) and MSV000095635 (in MassIVE; last accessed on Aug 18th, 2024). The cryo-EM density maps have been deposited in the RCSB Protein Data Bank (PDB; https://www.rcsb.org) with accession code EMD-61187 [https://www.ebi.ac.uk/emdb/EMD-61187]. Raw data from all native MS measurements are available in Supplementary Data 1. Source data are provided with this paper.

Research involving human participants, their data, or biological material

Policy information about studies with human participants or human data. See also policy information about sex, gender (identity/presentation), and sexual orientation and race, ethnicity and racism.

Reporting on sex and gender	4 male human participants donated their sera for experiments on natural haptoglobin.
Reporting on race, ethnicity, or other socially relevant groupings	All 4 participants are adult Han Chinese males.
Population characteristics	Participants were not previously diagnosed with any health conditions relevant to this study. Age range: 25 - 48.
Recruitment	All participants are co-authors of this manuscript.
Ethics oversight	The blood samples utilized in this research were donated by four volunteers, from whom written informed consent was obtained, in accordance with Peking University People's Hospital Ethical Regulations for Research Involving Human Subjects (#2023PHB273-001).

Note that full information on the approval of the study protocol must also be provided in the manuscript.

Field-specific reporting

Please select the one below that is the best fit for your research. If you are not sure, read the appropriate sections before making your selection.

☒ Life sciences ☐ Behavioural & social sciences ☐ Ecological, evolutionary & environmental sciences

For a reference copy of the document with all sections, see nature.com/documents/nr-reporting-summary-flat.pdf

Life sciences study design

All studies must disclose on these points even when the disclosure is negative.

Sample size	All samples tested in this work were employed for method development or proof of concept. Four sera samples were sufficient for demonstration of the performance of the developed methods.
Data exclusions	No data were excluded.
Replication	At least three replicates were measured for quantitative evaluation of the performance of the developed purification method. For the sera samples, three different methods were employed for cross-validation.
Randomization	In comparison of different methods, the same model protein system was employed for evaluation of each aspect of performance, so no randomization was needed.
Blinding	Blinding was not relevant to this study because no group allocation was made.

Reporting for specific materials, systems and methods

We require information from authors about some types of materials, experimental systems and methods used in many studies. Here, indicate whether each material, system or method listed is relevant to your study. If you are not sure if a list item applies to your research, read the appropriate section before selecting a response.

Materials & experimental systems

n/a	Involved in the study
☐	☒ Antibodies
☒	☐ Eukaryotic cell lines
☒	☐ Palaeontology and archaeology
☒	☐ Animals and other organisms
☒	☐ Clinical data
☒	☐ Dual use research of concern
☒	☐ Plants

Methods

n/a	Involved in the study
☒	☐ ChIP-seq
☒	☐ Flow cytometry
☒	☐ MRI-based neuroimaging

Antibodies

Antibodies used	commercial Monoclonal Anti-Green Fluorescent Protein (GFP) antibody produced in mouse (G6539) acquired from Sigma-Aldrich.
Validation	According to the Certificate of Analysis provided by the manufacturer, the antibody was tested with radial immunodiffusion and western blot. The references are available at the website: https://www.sigmaaldrich.com/SG/en/search/g6539?focus=papers&page=1&perpage=30&sort=relevance&term=G6539&type=citation_search

Plants

Seed stocks	*Report on the source of all seed stocks or other plant material used. If applicable, state the seed stock centre and catalogue number. If plant specimens were collected from the field, describe the collection location, date and sampling procedures.*
Novel plant genotypes	*Describe the methods by which all novel plant genotypes were produced. This includes those generated by transgenic approaches, gene editing, chemical/radiation-based mutagenesis and hybridization. For transgenic lines, describe the transformation method, the number of independent lines analyzed and the generation upon which experiments were performed. For gene-edited lines, describe the editor used, the endogenous sequence targeted for editing, the targeting guide RNA sequence (if applicable) and how the editor was applied.*
Authentication	*Describe any authentication procedures for each seed stock used or novel genotype generated. Describe any experiments used to assess the effect of a mutation and, where applicable, how potential secondary effects (e.g. second site T-DNA insertions, mosiacism, off-target gene editing) were examined.*
